# Radiochemotherapy combined with NK cell transfer followed by second-line PD-1 inhibition in a patient with NSCLC stage IIIb inducing long-term tumor control: a case study

**DOI:** 10.1007/s00066-019-01434-9

**Published:** 2019-02-11

**Authors:** Konrad Kokowski, Stefan Stangl, Sophie Seier, Martin Hildebrandt, Peter Vaupel, Gabriele Multhoff

**Affiliations:** 1 Pneumology and Pneumologic Oncology, Klinikum Bogenhausen, Munich, Germany; 2 Radiation Immuno-Oncology, Center for Translational Cancer Research TUM (TranslaTUM), Einsteinstr. 25, 81675 Munich, Germany; 3 TUMCells, TUM School of Medicine, Munich, Germany; 4 Department of Radiation Oncology, Klinikum rechts der Isar, TU München (TUM), Munich, Germany; 5 Institute of Innovative Radiotherapy (iRT), Department of Radiation Sciences (DRS), Helmholtz Center Munich (HMGU), Munich, Germany; 6 Partner site Munich, Deutsches Konsortium für Translationale Krebsforschung (DKTK), Munich, Germany; 7 Institute of Clinical Chemistry and Pathobiochemistry, Klinikum rechts der Isar, TU München (TUM), Munich, Germany

**Keywords:** Membrane Hsp70, Radiotherapy, Lung cancer, Immune checkpoint inhibition, Adoptive NK cell transfer, Membran-Hsp70, Radiotherapie, Lungenkrebs, Immuncheckpoint-Inhibition, Adoptiver NK-Zelltransfer

## Abstract

**Background:**

Membrane heat shock protein 70 (mHsp70) is indicative of high-risk tumors and serves as a tumor-specific target for natural killer (NK) cells stimulated with Hsp70 peptide (TKD) and Interleukin(IL)-2. Radiochemotherapy (RCT), mHsp70-targeting NK cells, and programmed death(PD)-1 inhibition were combined to improve the efficacy of tumor-specific immune cells in a non-small cell lung carcinoma (NSCLC) patient.

**Patient:**

Following simultaneous RCT (64.8 Gy), a patient with inoperable NSCLC (cT4, cN3, cM0, stage IIIb) was treated with 4 cycles of autologous ex vivo TKD/IL-2-activated NK cells and the PD-1 antibody nivolumab as a second-line therapy. Blood samples were taken for immunophenotyping during the course of therapy.

**Results:**

Adoptive transfer of ex vivo TKD/IL-2-activated NK cells after RCT combined with PD-1 blockade is well tolerated and results in superior overall survival (OS). No viable tumor cells but a massive immune cell infiltration in fibrotic tissue was detected after therapy. Neither tumor progression nor distant metastases were detectable by CT scanning 33 months after diagnosis. Therapy response was associated with significantly increased CD3^−^/NKG2D^+^/CD94^+^ NK cell counts, elevated CD8^+^ to CD4^+^ T cell and CD3^−^/CD56^bright^ to CD3^−^/CD56^dim^ NK cell ratios, and significantly reduced regulatory T cells (Tregs) in the peripheral blood.

**Conclusion:**

A combined therapy consisting of RCT, mHsp70-targeting NK cells, and PD-1 antibody inhibition is well tolerated, induces anti-tumor immunity, and results in long-term tumor control in one patient with advanced NSCLC. Further, randomized studies are necessary to confirm the efficacy of this combination therapy.

## Zusammenfassung

**Hintergrund:**

Membran-Hsp70 (mHsp70) ist ein Biomarker für aggressive Tumoren, der als tumorspezifische Erkennungsstruktur für Hsp70-Peptid-(TKD-)/IL-2-aktivierte NK-Zellen dient. Radiochemotherapie (RCT), Hsp70-spezifische NK-Zellen und PD1-Inhibition wurden kombiniert, um die Effizienz tumorspezifischer Immuneffektorzellen in einem Patienten mit fortgeschrittenem NSCLC zu steigern.

**Patient:**

Nach simultaner RCT (64,8 Gy) und 4‑maliger Behandlung mit ex vivo TKD-/IL-2-aktivierten, autologen NK-Zellen wurde der Patient mit inoperablem NSCLC (cT4, cN3, cM0, Stadium IIIb) mit dem PD-1-Antikörper Nivolumab als Zweitlinientherapie behandelt. Blutproben für die Immuntypisierung wurden während des gesamten Therapieverlaufs gewonnen.

**Ergebnisse:**

Der adoptive Transfer von ex vivo TKD-/IL-2-aktivierten NK-Zellen nach RCT kombiniert mit einer PD-1-Blockade war gut verträglich und führte zu einem signifikant verlängerten Gesamtüberleben. Nach Therapie waren keine vitalen Tumorzellen, aber eine massive Infiltration von NK- und T‑Zellen im fibrotischen Tumorgewebe nachweisbar. Im letzten CT, 33 Monate nach Diagnosestellung, waren weder Tumorprogress noch Fernmetastasen nachweisbar. Das Tumoransprechen war mit einem signifikanten Anstieg von CD3^−^/NKG2D^+^/CD94^+^-NK-Zellen, erhöhten CD8^+^/CD4^+^-T-Zell und CD3^−^/CD56^bright^/CD3^−^/CD56^dim^-NK-Zellverhältnissen und mit signifikant reduzierten Zahlen an regulatorischen T‑Zellen im peripheren Blut assoziiert.

**Schlussfolgerung:**

Eine Kombinationstherapie bestehend aus RCT, Hsp70-aktivierten NK-Zellen und PD-1-Inhibition ist gut verträglich, induziert antitumorale Immunantworten und führt zu einem signifikant verlängerten Gesamtüberleben in einem Patienten mit fortgeschrittenem NSCLC. Weitere randomisierte Studien sind notwendig, um den Wert dieser Kombinationstherapie zu bestätigen.

## Introduction

Stress-inducible Hsp70 is frequently overexpressed in the cytosol and presented on the plasma membrane of high-risk tumors including locally advanced lung cancer and therefore serves as a universal tumor biomarker [1]. Despite combined treatment regimens consisting of radio- and (cisplatinum-based) chemotherapy (RCT), most patients with non-operable, advanced NSCLC show disease progression and poor overall survival [2–5]. Chronic inflammation, anti-apoptotic pathways, and nuclear factor kappa-light chain-enhancer of activated B cells(NFκB)-, hypoxia-inducible factor(HIF)-, and signal transducer and activator of transcription(STAT)- driven [6, 7] immunosuppressive mechanisms [8] can thwart anti-tumor immune responses. A major breakthrough has been the blockade of immune checkpoint inhibitors, including PD-1/PD-L1 (programnmed cell death ligand-1), providing inhibitory feedback loops for immune-mediated tumor rejection [9, 10]. In healthy individuals, checkpoint inhibitors prevent autoimmunity, whereas in cancer patients, they abrogate cytolytic and migratory activities of T and NK cells [11, 12]. Nivolumab, a fully humanized IgG4 antibody, targets PD-1 and thereby attenuates inhibitory signals [9, 11], resulting in objective tumor responses [13, 14]. In melanoma and glioblastoma cells, RCT has been found to upregulate PDL-1 expression [15]. Despite promising clinical results in NSCLC patients after PDL-1 antibody therapy [10], a relevant proportion of patients do not respond to therapy. This might be partly due to the absence of anti-tumor-specific effector cells. Therefore, anti-Hsp70-activated NK cells were combined with anti-PD-1 inhibition in a patient with advanced NSCLC after RCT.

## Methods

### Ethics, patient characteristics, therapies

Written informed consent was obtained from the patient and the clinical trial protocol (NSCLC-TKD/IL-2 EudraCT-No.: 2008-002130-30) was approved by the institutional ethical review board of the Klinikum rechts der Isar, TU München (TUM). A 58-year-old male smoker was diagnosed with inoperable, stage IIIb squamous NSCLC (cT4, cN3, cM0; Karnofsky >90%) in 11/2015. The patient was treated with simultaneous cisplatinum/vinorelbine-based RCT (11/2015–02/2016) with a total radiation dose of 64.8 Gy (single fractions of 1.8 Gy). Following RCT and CT scanning, the patient received 4 cycles of ex vivo TKD/IL-2-stimulated, autologous NK cells (3/2016–6/2016) on a monthly basis. Sixteen months after diagnosis (3/2017–4/2017), the patient received 3 cycles nivolumab (Bristol-Myers Squibb, Princeton, NJ, USA; 3 mg/kg body weight, total dose 200 mg), as second-line therapy. Blood samples were taken between 0 and 20 months (V0, diagnosis; V1, CT after RCT; V2, NK cell therapy, V3–V5, CT after RCT and NK cell therapy; V6, nivolumab therapy; V7, CT-guided bronchoscopy). Radiographic responses of the tumor were staged according to RECIST1.1 criteria.

### Ex vivo stimulation of NK cells with TKD/IL-2

Following RCT and CT scanning, leukocyte concentrates were obtained by a 3–4 h leukapheresis (Cobe Spectra, Heimstetten, Germany) at the University Hospital Regensburg, Germany. PBLs were isolated by density gradient centrifugation in a closed SEPAX system (Biosafe, Eysins, Switzerland) and resuspended in CellGro SCGM stem cell medium (CellGenix, Freiburg/Breisgau, Germany). After counting, 5–10 × 10^6^ PBL/ml in CellGro medium were incubated with 2 µg/ml GMP-grade TKD peptide and 100 IU/ml recombinant IL-2 (Proleukin, Novartis, Nürnberg, Germany) [16], transferred into 250 ml Teflon bags (Vue-Life-118, CellGenix, Freiburg/Breisgau, Germany), and cultured in an incubator (Heraeus, Nürnberg, Germany) under gentle rotation at 37 °C, 5% CO_2_, in a humidified atmosphere (90%) for 3–5 days in a GMP laboratory (TUM Cells, Munich, Germany) according to a protocol of a phase I/II clinical trial [17, 18]. Activated cells were harvested, washed twice, and resuspended in Ringer’s lactate solution (500 ml) substituted with 0.1% human serum albumin (HSA). Sterility testing of intermediate and end products was performed regularly before and after reinfusion. Between 12 and 24 h after cell preparation, cells were reinfused by iv injection within 30–60 min using a stem cell reinfusion set. Tumor staging was performed by computed tomography (CT) and/or positron-emission tomography PET/CT. Bronchoscopies were taken under CT-guidance.

### Laboratory parameters and flow cytometric analysis of peripheral blood lymphocytes

Routine laboratory parameters (differential blood counts, RBC parameters, white blood cell counts), blood chemistry (creatinine, AST/SGOT, ALT/SGPT, γ‑GT, LDH) were determined after each treatment and in the follow-up period. Lymphocyte subpopulations were measured by flow cytometry on a FACSCalibur instrument (BD Biosciences, San Jose, CA, USA) in the peripheral blood of the patient at the different visits (V0–V7) with different antibody combinations (Supplementary Table 1): CD3^−^/CD19^+^ B cells, CD3^+^ T cells, CD3^+^/CD4^+^ helper T cells, CD3^+^/CD8^+^ cytotoxic T cells, CD3^+^/CD56^+^ NKT cells, CD3^+^/CD4^+^/FoxP3^+^, CD3^+^/CD8^+^/FoxP3^+^ regulatory T cells, CD3^−^/NKG2D^+^ NK cells, CD3^−^/NKp30^+^ NK cells, CD3^−^/NKp46^+^ NK cells, CD3^−^/CD94^+^ NK cells, CD3^−^/CD56^bright^; and CD3^−^/CD56^dim^ NK cells.

**Table 1:** Number of re-infused total white blood cells (WBC), total lymphocytes, total CD3^−^/CD56^+^ NK cell counts, and percentage of lymphocytes and CD3^−^/CD56^+^, CD3^−^/CD56^dim^, and CD3^−^/CD56^bright^ NK cells at reinfusion cycle 1 to 4. Viability of the reinfused cells was always >92%

Cycle	1	2	3	4
WBC (× 10^9^)	1.2	2.8	3.8	3.2
Total lymphocytes (× 10^9^)	1.2	2.5	3.5	2.9
Total CD3^−^/CD56^+^ NK cells (× 10^8^)	1.7	3.5	5.3	4.0
CD3^−^/CD56^+^ NK cells (%; normal range: 5–35%)	10	14	15	16
CD3^−^/CD56^dim^ NK cells (%)	2	3	2	3
CD3^−^/CD56^bright^ NK cells (%)	9	11	13	13

### Measurement of serum Hsp70 levels

Serum (S-Monovette 7.5 ml Z, Sarstedt, Nürnbrecht, Germany) was obtained after centrifugation of peripheral blood (10 min at 4000 rpm). Aliquots (300 µl) were prepared and directly stored at −80 °C. Hsp70 serum concentrations were determined using the lipHsp70 ELISA [20] and the commercial Duo Set ELISA-kit (R&D Systems, Wiesbaden, Germany) following the manufacturer’s instructions.

### Immunohistochemistry (IHC)

For IHC, formalin-fixed, paraffin-embedded (FFPE) specimens (4 µm) were stained on a Ventanas Benchmark XT using CD3- and CD56-specific antibodies (BD).

### Statistics

The Student’s *t-*test was used since the data were normally distributed. In all experiments, differences were considered as statistically significant at a value *p* < 0.05.

## Results

### Clinical responses of a patient with NSCLC to combined NK cell and nivolumab therapy after RCT

**Fig. 1:**
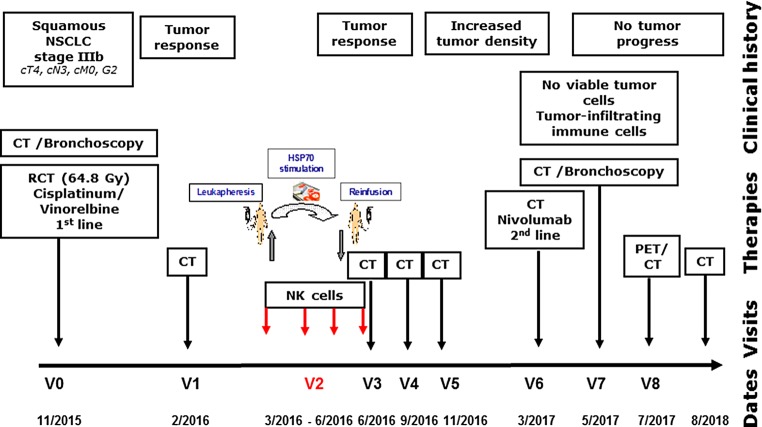
Schematic representation of the clinical history, therapy, and visits of a patient diagnosed with squamous NSCLC (stage IIIb, cT4, cN3, cM0, G2) in 11/2015 (V0). After simultaneous RCT (cisplatinum/vinorelbine, 64.8 Gy), a partial tumor response was evidenced by CT scanning (V1). Four weeks later (V2, 3/2016), the patient received 4 cycles of ex vivo TKD/IL-2-activated, autologous NK cells on a monthly basis. After 3 restagings on a three-monthly basis (V3–V5) without tumor progression, an increased cell density was detected in the right central lobe of the lung in 3/2017 (V6). After 3 injections of nivolumab (3/2017–4/2017) as a second-line therapy, a CT-guided bronchoscopy was performed (V7, 5/2017). No viable tumor cells but a massive immune cell infiltration was detected in the fibrotic tumor (V7). CT-based restagings 3 months later (V8, 7/2017) and 35 months after diagnosis did not reveal tumor progression or distant metastasis

**Fig. 2:**
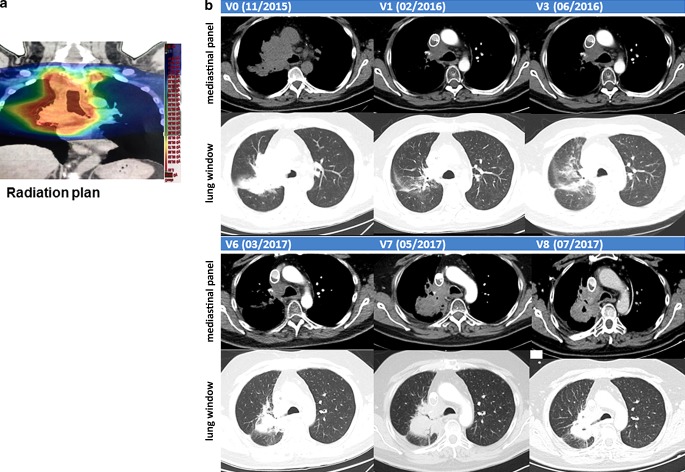
**a** Radiation plan. **b** Representative computed tomograpgy (CT) scans (upper panel: mediastinal window; lower panel: lung window) before and after therapies (V0–V8): lung at diagnosis (V0, 11/2015), after RCT (V1, 2/2016), after NK cell therapy (V3, 3/2016), after pseudo-tumor progress (V6, 3/2017), after 3 injections of nivolumab treatment (V7, 5/2017), and 20 months after diagnosis without tumor progression (V8, 7/2017)

**Fig. 3:**
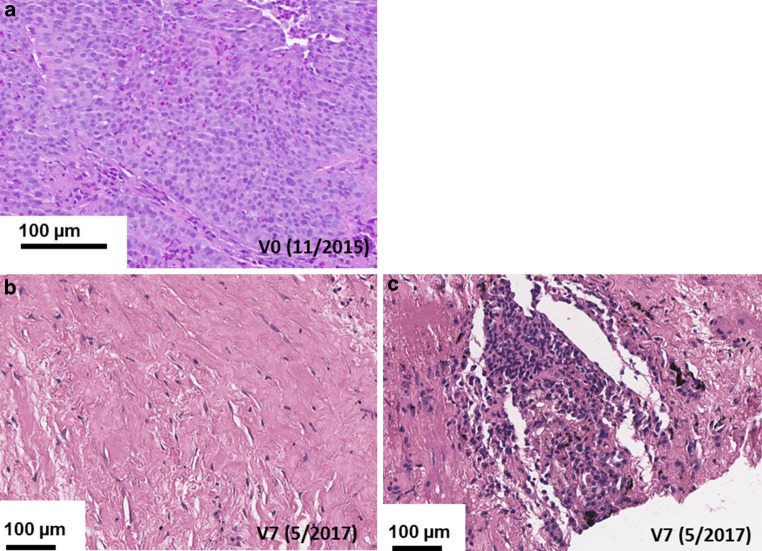
Immunohistochemistry (IHC) analysis of CT(computed tomography)- guided bronchoscopies at V0 and V7 of the NSCLC patient. Representative Hematoxylin and Eosin (H&E) stained tumor section. **a** Biopsy was taken during CT-guided bronchoscopy at diagnosis (V0, 11/2015). **b** Representative H&E stained fibrotic tumor section. Biopsy was taken during CT-guided bronchoscopy after radiochemotherapy (RCT), natural killer (NK) cell, and nivolumab therapy (V7, 5/2017). **c** Representative H&E stained fibrotic tumor section. Biopsy was taken during CT-guided bronchoscopy after RCT, NK cell, and nivolumab therapy (V7, 5/2017). Scale bar, 100 µm

A 58-year-old male patient was diagnosed with histologically proven, inoperable, stage IIIb squamous NSCLC (cT4, cN3, cM0; Karnofsky >90%) in 11/2015 (V0; Fig. 1). A mHsp70-positive tumor phenotype (V0) was confirmed by elevated exosomal Hsp70 (exHsp70) serum levels (11 ± 2.4 ng/ml), as determined by the lipHsp70 ELISA [20]. The radiation plan (total dose: 64.8 Gy) is shown in Fig. 2a. H&E staining of a bronchoscopy at diagnosis (V0) revealed a typical tumor morphology with atypical mitoses (Fig. 3a). Following simultaneous RCT, a partial tumor response was assessed after 3 months by CT scanning (V1, Fig. 2b). The RCT-induced decrease in tumor size was accompanied by a drop in exHsp70 concentrations (11 ± 2.4 ng/ml to 8 ± 2.3 ng/ml to 6 ± 1.1 ng/ml, V0–V2). The amount of free Hsp70, which predominantly originates from dying tumor cells (R&D ELISA), remained unaltered between V0 and V1, but gradually increased thereafter.

**Fig. 4:**
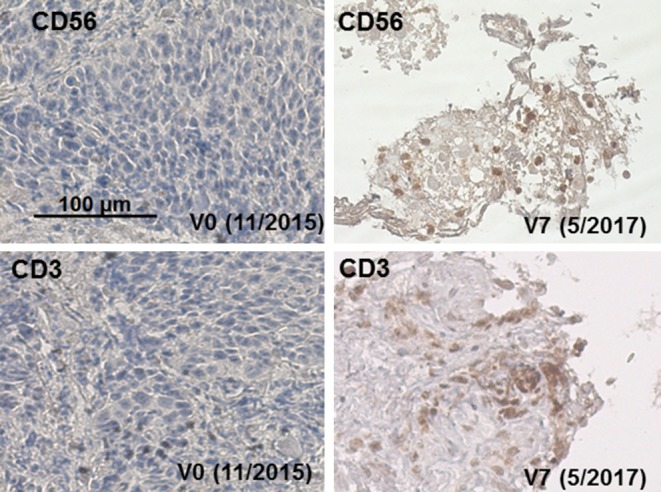
Representative IHC staining of tumor sections at V0 (11/2015) and V7 (5/2017) using antibodies directed against CD56 and CD3. Biopsies were taken during CT-guided bronchoscopy at diagnosis and after RCT, NK cell, and nivolumab therapy (V7, 5/2017)

Four weeks after the end of RCT, the patient received 4 iv injections of ex vivo TKD/IL-2-stimulated NK cells (V2, 3/2016–6/2016) every following month. The therapy was well tolerated, as demonstrated earlier in a phase I and a running phase II clinical trial [17, 18]. Previous data demonstrated that stimulation of PBL with TKD/IL-2 selectively stimulates CD3^−^ NK cells but not CD3^+^ T cells [16–18]. NK cell activation was found to be superior when non-sorted PBL instead of sorted NK cells were used for stimulation [18, 21]. The number of re-infused lymphocytes and NK cells increased from 1.2 to 2.9 × 10^9^ and 1.7 to 4.0 × 10^8^, respectively, between the first and fourth reinfusion cycle, and CD56^bright^ cells dominated over CD56^dim^ NK cells (Table 1).

CT scanning after NK therapy (V3–V5) revealed no tumor progression 12 months after diagnosis (data not shown). As Hsp70 serum concentrations increased concomitantly with C‑reactive protein (CRP) between V2 and V4 (exHsp70: 22 ± 2.6 ng/ml), it was assumed that inflammation rather than tumor growth caused this increase. Sixteen months after diagnosis (V6), a higher cell density was detected by CT scanning in the previously irradiated area of the right upper lobe of the lung (Fig. 2b). Since tumor progression was assumed based on CT scanning, the patient received nivolumab as a second-line therapy in a non-metastatic setting. However, 18 months after diagnosis (V7), IHC analysis of a CT-guided bronchoscopy revealed no signs of viable tumor cells, but fibrotic tissue which is indicative of a pseudo-progress (V7, Fig. 3b). We hypothesize that the massive infiltration of CD56^+^ NK and CD3^+^ T cells detected inside the tumor (V7) which was absent at diagnosis (V0, Fig. 4) was responsible for the increased cell density determined by CT scanning at V6. In the follow-up, no tumor progression (V8, Fig. 2b) and no distant metastasis were detectable by CT scanning 33 months after diagnosis.

Apart from a transient increase in CRP (V3–V4) and after nivolumab treatment (V6–V7), routine laboratory parameters remained within normal ranges throughout the whole therapy (data not shown).

### Phenotypic characterization of patient-derived effector cells

**Fig. 5:**
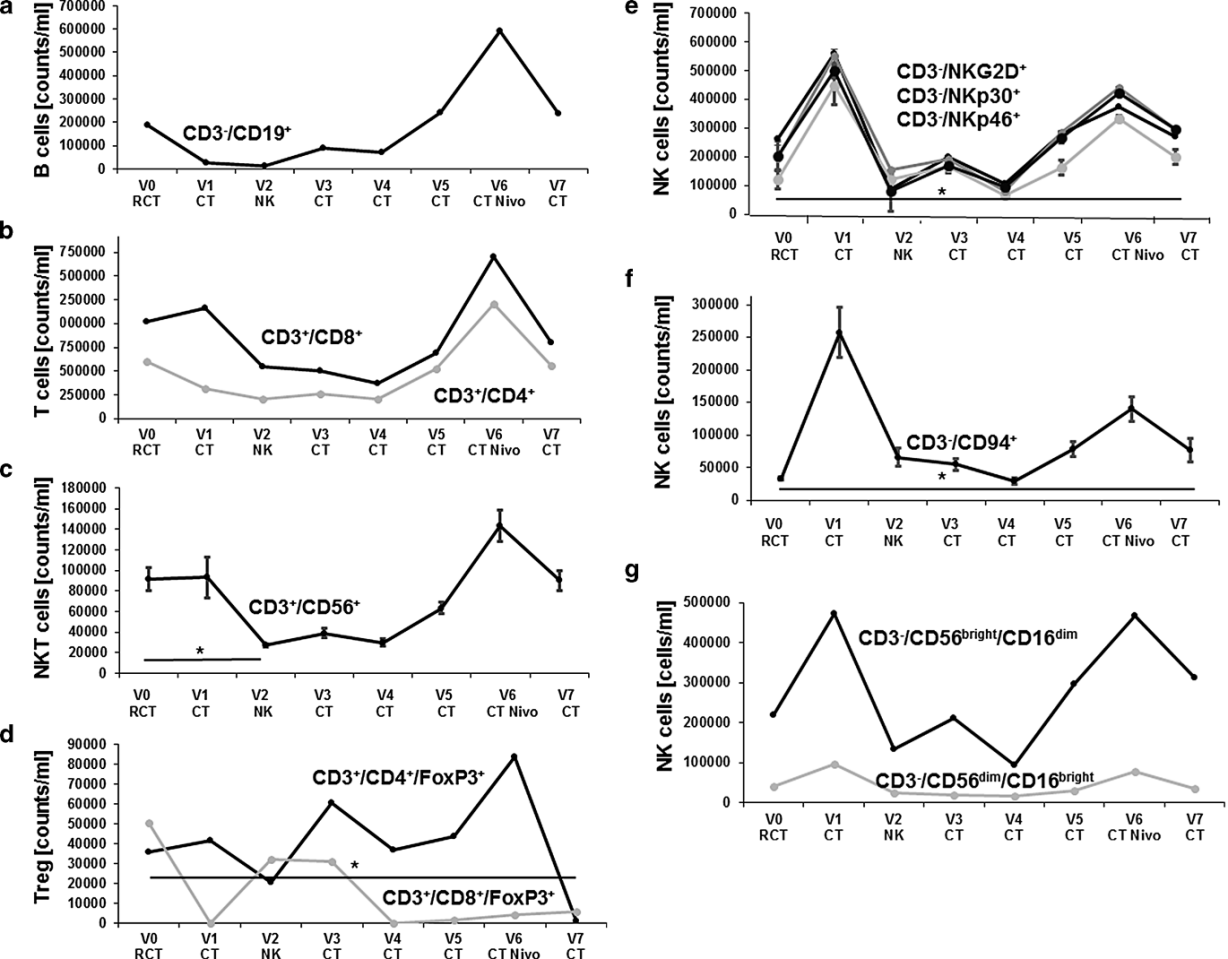
Absolute counts/ml of different lymphocyte subpopulations at diagnosis (V0), after therapy, and in the follow-up period (V1–V7). **a** CD19^+^ B cells at diagnosis (V0), after RCT (V1), after 4 cycles of NK cell therapy (V2), after 3‑monthly CT-guided restaging (V3–V5), and upon 3 cycles of nivolumab treatment and CT-guided bronchoscopy (V7). **b** CD3^+^/CD8^+^ cytotoxic T cells and CD3^+^/CD4^+^ helper T cells at diagnosis (V0), after RCT (V1), after 4 cycles of NK cell therapy (V2), after 3‑monthly CT-guided restaging (V3–V5), and upon 3 cycles of nivolumab treatment and CT-guided bronchoscopy (V7). **c** CD3^+^/CD56^+^ NK-like T (NKT) cells at diagnosis (V0), after RCT (V1), after 4 cycles of NK cell therapy (V2), after 3‑monthly CT-guided restaging (V3–V5), and upon 3 cycles of nivolumab treatment and CT-guided bronchoscopy (V7). *p < 0.05. **d** Immunosuppressive CD4^+^/FoxP3^+^ and CD8^+^/FoxP3^+^ regulatory T cells (Treg) at diagnosis (V0), after RCT (V1), after 4 cycles of NK cell therapy (V2), after 3‑monthly CT-guided restaging (V3–V5), and upon 3 cycles of nivolumab treatment and CT-guided bronchoscopy (V7). **e** CD3^−^/NKG2D^+^, CD3^−^/NKp30^+^, CD3^−^/NKp46^+^ NK cell subpopulations at diagnosis (V0), after RCT (V1), after 4 cycles of NK cell therapy (V2), after 3‑monthly CT-guided restaging (V3–V5), and upon 3 cycles of nivolumab treatment and CT-guided bronchoscopy (V7). **f** CD3^−^/CD94^+^ NK cells at diagnosis (V0), after RCT (V1), after 4 cycles of NK cell therapy (V2), after 3‑monthly CT-guided restaging (V3–V5), and upon 3 cycles of nivolumab treatment and CT-guided bronchoscopy (V7). **g** CD3^−^/CD56^bright^/CD16^dim^ and CD3^−^/CD56^dim^/CD16^bright^ NK cell subpopulations at diagnosis (V0), after RCT (V1), after 4 cycles of NK cell therapy (V2), after 3‑monthly CT-guided restaging (V3–V5), and upon 3 cycles of nivolumab treatment and CT-guided bronchoscopy (V7). *p < 0.05. Mean values ± SD

The composition of major lymphocyte subpopulations was determined between V0 and V7 in the peripheral blood. The number of B cells, which was below the normal range at diagnosis (V0), further dropped after RCT, recovered in the follow-up period after NK cell therapy (V5), and further increased above baseline levels after nivolumab treatment (V6, Fig. 5a). The patient exhibited elevated CD8^+^/CD4^+^ ratios throughout the course of therapy (Fig. 5b). The significant decrease in CD3^+^/CD56^+^ cell counts after RCT/NK cell therapy (V0–V2, *p* < 0.05) reached initial levels following nivolumab therapy (V6, Fig. 5c). Immunosuppressive regulatory CD4^+^/CD8^+^ Tregs (Fig. 5d) transiently increased after NK (V3) and nivolumab therapy (V6) but dropped more than 10-fold at V7. The absolute numbers of CD3^−^ NK cell subsets such as CD3^−^/NKG2D^+^, CD3^−^/NKp30^+^, and CD3^−^/NKp46^+^, which are assumed to be involved in the killing of mHsp70 [16], transiently increased after RCT (V1), dropped thereafter, increased to above initial levels after nivolumab therapy (V6), and remained significantly elevated compared to V0 thereafter (*p* < 0.05; V7; Fig. 5e and f). The number of CD3^−^/CD94^+^ NK cells was raised about 10-fold after RCT and 3‑fold after nivolumab therapy compared to initial levels. The resistance of NK cells to RCT might be due to elevated intracellular glutathione levels [20]. CD3^−^/CD56^bright^ NK cells which were above that of CD3^−^/CD56^dim^ NK cells play a central role in the recognition of mHsp70 positive tumor cells ([21]; Fig. 5g). It appears that nivolumab is supportive for CD3^−^/CD56^bright^ NK cells because the number of this NK cell subset nearly doubled after nivolumab therapy (Fig. 5g). In contrast, the number of the CD3^−^/CD56^dim^ NK cell subpopulation remained nearly unaltered throughout the therapy (Fig. 5g).

**Fig. 6:**
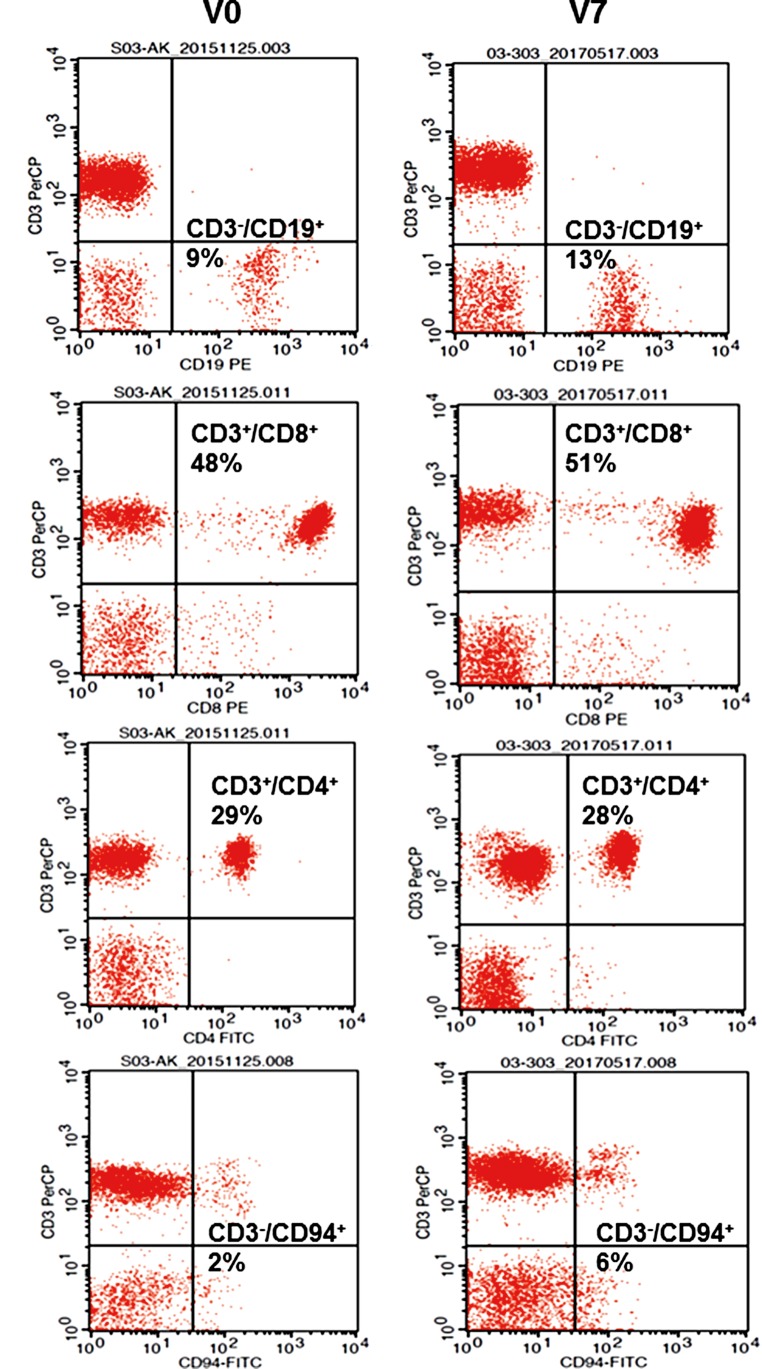
Representative dot blot analysis of selected major lymphocyte subpopulations such as CD3^−^/CD19 ^+^ B cells, CD3^+^/CD8^+^ cytotoxic T cells, CD3^+^/CD4^+^ T helper cells, and CD3^−^/CD94^+^ NK cells at diagnosis (V0) and at the end of all therapies (V7) as determined by multiparameter flow cytometry. Percentages of positively stained cells are indicated in the respective graphs. Combinations of fluorescence-labelled antibodies used for flow cytometry are summarized in Supplementary Table 1

A comparison of representative flow cytometric dot plots of selected major lymphocyte subpopulations at V0 and V7, such as CD3^−^/CD19^+^ B cells (9% vs. 13%), CD3^+^/CD8^+^ cytotoxic lymphocytes (48% vs. 51%), CD3^+^/CD4^+^ T helper cells (29% vs. 28%), CD3^−^/CD94^+^ NK cells (2% vs. 6%), and CD3^−^/CD56^+^ NK cells (10% vs. 16%; Table 1). The data showed major differences in the percentage of positively stained cells and in the receptor densities as revealed by a shift of the respective plots along the x‑ and y‑axes (Fig. 6h).

## Discussion

A patient with advanced NSCLC was treated with ex vivo-stimulated NK cells and nivolumab after RCT for tumor debulking [22] and for increasing damage-associated molecular patterns (DAMPs) [23, 24] including Hsp70. Membrane Hsp70 serves as a tumor target for activated NK cells [1, 16–18, 21], whereas cytosolic Hsp70 impairs apoptosis [25]. Previously, exHsp70 has been shown to predict the mHsp70 status of primary tumors and viable tumor mass in tumor-bearing mice [26] and NSCLC patients [27]. Membrane Hsp70 positivity of the patient’s tumor was confirmed by elevated exHsp70 serum concentrations.

Free Hsp70 derived from dying cells is indicative of tumor response [19]. The concentration of free Hsp70 that gradually increases between V0 and V7 (0–18 months) most likely reflects tumor cell killing, whereas the drop in exHsp70 after RCT might be associated with a reduction in viable tumor mass. A transient Hsp70 increase which occurs concomitantly with that of CRP after NK and PD-1 treatment might be attributed to therapy-induced inflammation.

Historically, RCT has been considered as immunosuppressive because of large radiation fields that included substantial volumes of the blood and bone marrow [28]. However, due to advances in radiation planning and equipment, normal tissue damage can be minimized. In preclinical models, RCT has been shown to induce immunogenic tumor cell death that might elicit abscopal effects [24, 29]. A recent study on lung cancer [11] and squamous carcinoma of the head and neck [30] demonstrated that anti-PD-1 therapies are most efficient in tumors with a high mutational load. The smoking history of the patient and the RCT-induced release of DAMPs might partly explain the beneficial outcome of the patient.

Stage III NSCLC patients after RCT in the control arm of the PACIFIC trial showed tumor progression already after 5.6 months [10]. The median OS of NSCLC patients in stage IIIA/B ranged between 28.7 and 20.3 months following standard versus high-dose conformal RCT [31, 32]. As a comparison, the NSCLC patient who received RCT, NK cells, and PD-1 was progression free for more than 35 months.

Immunophenotyping of peripheral blood lymphocytes of tumor patients by multicolor flow cytometry has been found to be useful for predicting outcome and detecting immunomodulation by ionizing irradiation [33, 34]. Similar to studies with mammary and prostate carcinomas [35], RCT also negatively impacts on the B cell compartment of the NSCLC patient, due to the sensitivity of B cells towards radiotherapy [36]. B cells, which are important players in the cross-talk of the innate and adaptive immunity [37], recovered at V5 and increased during nivolumab treatment, concomitant with the number of CD3^+^/CD8^+^, CD3^+^/CD4^+^, and CD3^+^/CD56^+^ T cells that might also contribute to protective anti-tumor immunity.

Regulatory CD4^+^/CD25^+^/FoxP3^+^ T cells (Tregs) are well known to attenuate T and NK cell activity by secretion of anti-inflammatory suppressive cytokines that impede cytotoxicity and migratory capacity of effector cells. Generally, tumor patients have elevated Treg counts in the peripheral blood and tumor microenvironment [38], which is associated with adverse clinical outcome. In the NSCLC patient, RCT did not immediately influence Tregs and during NK cell therapy, a transient increase of this population was observed. This finding is most likely due to an RCT-induced inflammatory response which might induce the production of IL-2. However, after nivolumab therapy (V7), Tregs dropped to below initial levels. As shown in preclinical models, a depletion of Tregs could restore T and NK cell mediated anti-tumor immunity [39].

NK cells play a crucial role in first-line defense against cancer [40–43]. Apart from CD8^+^ cytotoxic T cells [44], studies have indicated that the OS of patients with oropharyngeal cancer positively correlates with high numbers of tumor-infiltrating CD3^−^/CD56^+^ NK cells [45] that recognize antigens via activatory receptors belonging to the C‑type lectin family [21, 46, 47]. The success of an immune checkpoint inhibitor blockade is dependent on anti-tumor-specific effector cells [48, 49]. Similar to a patient with colon cancer [21, 45, 50], the stimulation with TKD/IL-2 induced a shift towards the CD3^−^/CD56^bright^ NK cell subset which was further propagated by nivolumab.

Apart from a complex network of inhibitory/activatory receptors with immune tyrosine-based inhibitory/activatory motives (ITIM/ITAM) [51], the presence or absence of PD-1 receptors also determines the cytolytic function of activated NK cells [52]. Therefore, inhibition of the PD-1 pathway after adoptive transfer of ex vivo-stimulated NK cells might synergistically enhance their survival and cytolytic activity. Exosomal Hsp70, which has been shown to attract NK cells in vitro [53], might further stimulate their migratory and cytolytic activity against mHsp70-positive tumors.
